# Extended and Repeated Cytoreductive Surgery in Recurrent Uterine Leiomyosarcoma: A Narrative Review

**DOI:** 10.3390/cancers18132061

**Published:** 2026-06-25

**Authors:** Antonio Maccio, Manuela Neri, Valerio Vallerino, Sonia Nemolato, Elisabetta Pusceddu, Gabriele Sole, Paolo Albino Ferrari

**Affiliations:** 1 Department of Obstetrics and Gynecology and Gynecological Oncology, Azienda di Rilievo Nazionale ed Alta Specializzazione “G. Brotzu”, Piazza A. Ricchi 1, 09121 Cagliari, Italy; antoniopm.maccio@aob.it (A.M.); manuela.neri@aob.it (M.N.); valerio.vallerino@aob.it (V.V.); 2 Department of Oncological Surgery, Azienda di Rilievo Nazionale ed Alta Specializzazione “G. Brotzu”, Piazza A. Ricchi 1, 09121 Cagliari, Italy; gabriele.sole@aob.it; 3 Unit of Anatomic Pathology, Azienda di Rilievo Nazionale ed Alta Specializzazione “G. Brotzu”, Piazza A. Ricchi 1, 09121 Cagliari, Italy; sonia.nemolato@aob.it; 4 Anesthesia and Intensive Care Unit, Liver Transplantation Center, Azienda di Rilievo Nazionale ed Alta Specializzazione “G. Brotzu”, Piazza A. Ricchi 1, 09121 Cagliari, Italy; elisabetta.pusceddu@aob.it; 5 Department of Thoracic Surgery, Azienda di Rilievo Nazionale ed Alta Specializzazione “G. Brotzu”, Piazza A. Ricchi 1, 09121 Cagliari, Italy

**Keywords:** uterine leiomyosarcoma, recurrent disease, cytoreductive surgery, metastasectomy, multivisceral resection, pulmonary metastasectomy, narrative review, gynecologic oncology

## Abstract

Background/Objectives: Recurrent uterine leiomyosarcoma (ULMS) frequently poses a surgical question because systemic options remain limited and recurrence patterns are heterogeneous. We reviewed the published evidence on repeated and extended cytoreductive surgery for recurrent ULMS, focusing on selection criteria, operative boundaries, and the role of multivisceral, thoracic, and peritoneal-directed procedures. Methods: This narrative review synthesizes peer-reviewed literature on surgically managed recurrent or metastatic ULMS, prioritizing contemporary guidelines, retrospective cohorts, pooled analyses, selected systematic reviews when directly relevant to the surgical question, and published illustrative reports. The search covered records available from database inception through 14 May 2026 and used PubMed/MEDLINE, Web of Science Core Collection, Scopus, Google Scholar, selected publisher databases, and citation-linked records. No new patient-level or institution-specific clinical data are presented. Results: The available evidence is entirely retrospective and strongly affected by selection bias, yet it consistently suggests that the best outcomes are observed when complete gross resection is feasible. Across published series, favorable features include isolated or limited recurrence, longer time to relapse, compartmentalized disease, lung-only metastases, and preserved performance status. Contemporary reports also show that repeat surgery may evolve into extensive multivisceral procedures involving bowel resection, upper-abdominal dissection, urinary tract reconstruction, diaphragmatic resection, and thoracic surgery. Peritoneal-directed CRS/HIPEC-type strategies remain supported mainly by small heterogeneous studies and a ULMS-specific systematic review, reinforcing feasibility but not routine use. Published illustrative reports confirm that serial metastasectomies can occasionally support prolonged survival in exceptional patients, but they cannot establish effectiveness. Conclusions: In highly selected patients, repeated and even extensive cytoreductive surgery may remain a rational disease-control strategy for recurrent ULMS. The central unmet need is not proof that surgery can work in exceptional cases, but better criteria to identify who benefits from iterative resection and when escalation to multivisceral or thoracoabdominal surgery is justified.

## Simple Summary

Uterine leiomyosarcoma is a rare and highly aggressive cancer that often returns after initial treatment. When recurrence remains technically removable, surgeons may consider repeated operations or extended cytoreduction, but the available evidence is scattered and mostly retrospective. This narrative review summarizes current data on recurrent uterine leiomyosarcoma treated surgically, with special attention to repeated metastasectomy, multivisceral cytoreduction, and procedures involving the bowel, upper abdomen, urinary tract, diaphragm, or thorax. Published cohort studies and illustrative reports suggest that complete gross resection can be meaningful in carefully selected patients, although this apparent benefit is strongly influenced by selection bias. Validated selection criteria and prospective collaborative data remain lacking.

## 1. Introduction

Uterine leiomyosarcoma (ULMS) is an uncommon but highly aggressive uterine mesenchymal malignancy with a marked tendency toward early hematogenous and intraperitoneal dissemination, frequent recurrence, and poor long-term survival [1,2].

Even after apparently localized treatment, recurrent disease is common and salvage strategies are constrained by the biological heterogeneity of relapse and the limited durability of currently available systemic therapies [1,3,4,5,6].

Current guidelines and expert reviews agree that surgery should be reconsidered whenever recurrence is technically resectable and complete gross resection appears feasible within a multidisciplinary framework [3,4,7,8].

At the same time, the evidence base remains weak because it is dominated by retrospective cohorts, mixed uterine sarcoma series, pooled analyses, and isolated single-patient reports rather than prospective comparative trials [7,8,9].

Accordingly, the clinical question is not whether surgery can ever produce long survivorship in recurrent ULMS, but which patients may benefit from repeated and increasingly extended cytoreduction as recurrence pattern, disease distribution, and operative complexity evolve over time [3,7,8,9,10,11,12,13,14,15,16,17].

This narrative review examines the published evidence on repeated metastasectomy and extended cytoreductive surgery in recurrent ULMS, with special attention to multivisceral procedures, thoracoabdominal disease, peritoneal-directed approaches, and the practical boundaries of surgical escalation [3,7,8,9,10,11,12,13,14,15,16,17].

Unlike broader reviews of uterine sarcoma management, it deliberately focuses on the iterative surgical trajectory that may occur after ULMS recurrence: repeated metastasectomy, escalation to multivisceral abdominopelvic cytoreduction, and integration of thoracic or peritoneal-directed procedures when complete gross resection remains plausible [3,7].

The objective is to synthesize the evidence relevant to patient selection, operative extent, compartment-specific surgical strategies, and the methodological limitations that should guide interpretation of reported survival benefits.

## 2. Materials and Methods

### 2.1. Review Design and Reporting Framework

This manuscript is a narrative review of the previously published literature and does not present new patient-level or institution-specific clinical material. Reporting was guided by the SANRA framework for narrative reviews, with explicit definition of the review aim, search strategy, eligibility criteria, evidence categories, interpretive approach, and limitations [18]. Because the aim was narrative synthesis rather than a formal systematic review, no quantitative pooling, meta-analysis, or formal risk-of-bias scoring was attempted. During manuscript preparation, ChatGPT (OpenAI, GPT-5.5 Thinking version) was used solely to assist with English grammar, syntax, and language polishing. The tool was not used to generate scientific content, perform literature searches, select references, analyze data, draw conclusions, or create figures or tables. All AI-assisted language suggestions were critically reviewed and edited by the authors, who take full responsibility for the final content of the manuscript.

### 2.2. Search Strategy and Coverage

The bibliographic search covered records available from database inception through 14 May 2026. The sources searched were PubMed/MEDLINE, Web of Science Core Collection, Scopus, Google Scholar, and selected publisher databases relevant to the topic, including ScienceDirect, SpringerLink, Wiley Online Library, Taylor & Francis Online, and MDPI. Citation-linked records from included articles and relevant guideline documents were also screened. Search terms were combined using variants of: “uterine leiomyosarcoma”, “ULMS”, “recurrent”, “metastatic”, “secondary cytoreduction”, “cytoreductive surgery”, “metastasectomy”, “pulmonary metastasectomy”, “peritoneal sarcomatosis”, “HIPEC”, and “morcellation”. No lower date limit was applied. No language restriction was imposed at the search stage; however, studies were included in the synthesis only when the available text provided sufficient extractable information on the surgical question.

### 2.3. Eligibility Criteria and Study Selection

Eligible sources included contemporary guidelines, expert reviews, retrospective or multi-institutional cohorts, registry-based analyses, pooled analyses, systematic reviews, and published illustrative reports addressing surgically managed recurrent or metastatic ULMS. Mixed uterine sarcoma, uterine malignancy, or leiomyosarcoma series were retained only when they directly informed a compartment-specific surgical question, such as pulmonary metastasectomy or peritoneal-directed cytoreduction, and their histologic heterogeneity was explicitly considered in interpretation. Studies were excluded when they did not address recurrent or metastatic disease, did not include a surgical-management component, reported only nonsurgical systemic therapy or radiotherapy without relevance to surgical decision making, were preclinical or non-peer-reviewed, duplicated data without adding relevant surgical information, or lacked sufficient clinical detail for narrative extraction.

Study selection was performed narratively according to relevance to the predefined surgical questions. Priority was given to ULMS-specific data and to sources reporting patient selection, complete gross resection or residual disease, anatomic site of recurrence, operative extent, survival outcomes, morbidity, and follow-up or survival time horizon. When evidence types differed, guideline-level sources were used to frame accepted clinical principles; retrospective ULMS-specific cohorts were used to summarize association signals between surgery and outcomes; pooled analyses and systematic reviews were used to contextualize broader leiomyosarcoma or peritoneal-directed evidence; and case reports were used only to illustrate technical feasibility boundaries rather than treatment effectiveness.

### 2.4. Evidence Mapping and Lightweight Appraisal

The included evidence was organized into four narrative domains: rationale for surgery in recurrent ULMS, cohort evidence for cytoreduction or metastasectomy, compartment-specific evidence, and selection factors. For cohort studies and surgical series, a lightweight structured appraisal extracted, when available, study design, population, sample size, disease setting, sites of recurrence or metastasis, selection features, complete gross resection or residual disease, reported follow-up or survival time horizon, morbidity, and main limitations. This approach was intended to improve transparency without implying the level of reproducibility or bias control expected from a formal systematic review.

**Figure 1:**
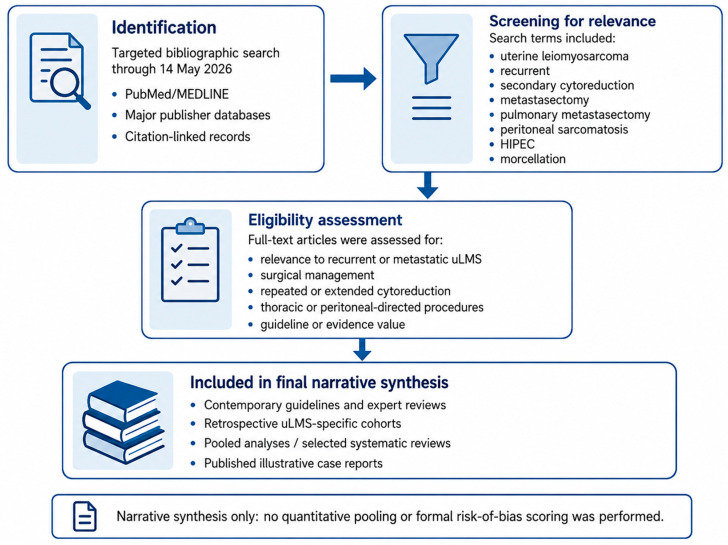
Evidence-mapping workflow for the narrative review of repeated and extended cytoreductive surgery in recurrent uterine leiomyosarcoma.

Figure 1 summarizes the evidence-mapping workflow used for the review. Because the review was designed as a narrative synthesis rather than a systematic review, the diagram should be interpreted as an evidence map rather than a PRISMA flow chart.

Evidence was interpreted according to disease specificity, surgical relevance, methodological robustness, consistency across independent datasets, and susceptibility to selection bias. This distinction is essential because highly selected surgical series and case reports can demonstrate what is technically possible, but they cannot establish causal treatment benefit [7,9,10,11,12,13,14,15,16,17,19,20,21,22,23,24,25,26,27,28,29].

## 3. Results

### 3.1. Rationale: Why Recurrent ULMS Remains a Surgical Question

ULMS differs from epithelial gynecologic malignancies because recurrence is often dominated by discrete metastatic deposits rather than diffuse chemosensitive peritoneal carcinomatosis, making local control a recurring issue in selected patients [1,3,4,7,8].

The therapeutic background is also important: although anthracycline-based chemotherapy, gemcitabine/docetaxel-based regimens, trabectedin, pazopanib, and other systemic approaches have activity, durable complete responses remain uncommon in metastatic or recurrent disease [5,6].

For that reason, surgical resection continues to have conceptual appeal whenever the burden of recurrence is anatomically limited, technically approachable, and potentially reducible to no gross residual disease [3,4,7,8,9,10].

The term extended cytoreduction in recurrent ULMS should therefore be understood pragmatically as surgery that goes beyond simple lesion excision to encompass bowel resection, upper-abdominal dissection, peritonectomy, splenectomy, diaphragmatic resection, bladder or ureteral procedures, and thoracic metastasectomy when needed to achieve complete gross resection [7,8,9,10,11,12,13,14,15,16,17].

This concept is clinically relevant because the biology of recurrent ULMS is heterogeneous: some patients relapse rapidly with multifocal systemic progression, whereas others experience serial compartmentalized recurrences that remain surgically approachable over years [1,2,3,4,7,8,9,15,16,17].

Published studies repeatedly suggest that the patients most likely to derive value from surgery are those with a longer recurrence-free interval, limited disease distribution, preserved performance status, and a high preoperative probability of complete resection [9,10,11,12,13,14,15,16,17].

To place these surgical considerations within the framework of current clinical guidance, Table 1 summarizes the main guideline-based principles relevant to recurrent ULMS and indicates how they align with the retrospective surgical literature reviewed.

However, all of these associations are vulnerable to indication bias, because the decision to operate is itself driven by favorable disease biology and technical resectability [7,8,9,10,11,12,13,14,15,16,17].

Current guidelines and retrospective surgical cohorts are broadly concordant on one point: surgery may be considered when recurrent disease is technically resectable and complete gross resection appears feasible, but treatment must remain individualized according to recurrence pattern, disease-free interval, prior therapy, patient status, and expected morbidity [3,4,7,9,10,11,12,13,14,15,16,17].

The guidelines do not provide validated criteria for repeated multivisceral cytoreduction, pulmonary metastasectomy, or cytoreductive surgery/hyperthermic intraperitoneal (CRS/HIPEC) in recurrent ULMS, which is why retrospective cohort data and prospective registry development remain clinically important [3,4,7,9,10,11,12,13,14,15,16,17,19,20,21,22].

### 3.2. Cohort Evidence on Cytoreduction and Metastasectomy in Metastatic or Recurrent ULMS

The published cohort literature supports surgery mainly through consistent association signals rather than through randomized evidence, and a focused bibliographic update through 14 May 2026 did not identify any prospective trial specifically testing repeated cytoreduction in recurrent ULMS [7,8,9,10,11,12,13,14,15,16,17].

In patients who already had metastatic ULMS at first diagnosis, Leitao et al. reported that complete gross resection was associated with longer progression-free and overall survival than incomplete cytoreduction, thereby establishing the principle that operative completeness matters even in advanced presentation [10].

In the recurrent setting, Giuntoli et al. found that secondary cytoreduction, longer time to recurrence, and isolated recurrence were independently associated with improved disease-specific survival, which remains one of the foundational retrospective observations favoring surgery in selected patients [9].

Leitao et al. further demonstrated that resection of both pulmonary and extrapulmonary recurrences could be integrated into management of recurrent ULMS, thereby broadening the surgical frame beyond the pelvis and lower abdomen alone [11].

Representative retrospective series are summarized in Table 2 [9,10,11,12,13,14,15,16].

The most clinically informative contemporary retrospective cohorts are those by Cybulska et al., Bizzarri et al., Yuan et al., and Tunç et al., because they provide more recent ULMS-focused data on complete resection, recurrence pattern, survival outcomes, and the selection-dependent nature of secondary cytoreduction [12,13,14,15,16].

These studies also clarify that recurrent ULMS surgery is not confined to simple nodulectomy: bowel, bladder, upper-abdominal, and thoracic procedures were repeatedly required in order to accomplish complete resection [11,12,13,14,15,16].

At the same time, the same cohorts demonstrate the limits of generalization, because patients with diffuse or rapidly progressive disease were less likely to be offered surgery in the first place, and adjuvant therapy patterns were heterogeneous across studies [9,10,11,12,13,14,15,16].

### 3.3. Compartment-Specific Evidence

Recurrent ULMS frequently relapses in the abdomen and pelvis, where mesentery, bowel, peritoneum, upper abdomen, retroperitoneum, and urinary tract may all become part of the operative field during salvage surgery [7,8,9,10,11,12,13,14,15,16,17].

#### 3.3.1. Abdominopelvic and Multivisceral Surgery

In the multi-institutional series by Bizzarri et al., bowel surgery was required in half of the operated patients, bladder surgery in 18.4%, and upper-abdominal surgery in 28.9%, which underscores how often successful secondary cytoreduction becomes multivisceral rather than purely gynecologic [13].

Yuan et al. similarly reported that secondary cytoreduction in recurrent ULMS frequently extended beyond the pelvis and included thoracic, bowel, and other extra-pelvic procedures in the operated subgroup [15].

These observations align with older expert reviews stating that recurrent uterine sarcoma surgery must be individualized according to disease topography rather than limited by traditional organ-based specialty boundaries [7,8].

Importantly, extensive abdominopelvic surgery in recurrent ULMS should not be confused with indiscriminate aggressiveness, because the published benefit signal depends on the realistic expectation that complete gross resection can be achieved with acceptable morbidity [3,4,7,8,9,10,11,12,13,14,15,16,17].

The available literature therefore supports technical escalation when disease remains resectable, but not the routine use of radical multivisceral surgery in biologically uncontrolled or widely disseminated relapse [3,4,7,8,9,10,11,12,13,14,15,16,17].

#### 3.3.2. Pulmonary Metastasectomy and Serial Thoracic Resections

The thoracic compartment deserves separate consideration because the lung is one of the most frequent sites of metastatic dissemination in ULMS, and several studies suggest that pulmonary metastasectomy may be meaningful in selected patients [1,3,4,11,17,30,31,32,33,34,35,36].

Leitao et al. established early that pulmonary and extrapulmonary resection could both be incorporated into recurrent ULMS management, while later pulmonary metastasectomy series in uterine malignancy confirmed the importance of disease-free interval, number of metastases, absence or control of extrapulmonary disease, and complete resection [11,31,34,35,36].

In a series focused on isolated pulmonary metastatic leiomyosarcoma, Stork et al. reported a 5-year overall survival of 55% after diagnosis of lung metastases and no postoperative mortality among the metastasectomy subgroup, although outcomes worsened with older age, larger primary tumors, and five or more lung metastases [32].

Burt et al. also showed that repeated and aggressive pulmonary resections for leiomyosarcoma metastases can extend survival in highly selected patients (with limited or compartmentalized disease, longer disease-free interval, preserved performance status, acceptable cardiopulmonary and functional reserve, absence of rapidly progressive multifocal dissemination, and a high preoperative probability of complete gross resection with acceptable morbidity), supporting the concept that serial thoracic surgery may sometimes be justified when recurrence remains anatomically limited [33].

More recent uterine-malignancy pulmonary metastasectomy datasets reinforce the same selection-dependent message. Kanzaki et al. reported complete resection in 91% of 57 patients and a 5-year overall survival of 68.8% among completely resected patients, with disease-free interval of >24 months identifying better candidates [34]. Nobori et al. analyzed 319 pulmonary metastasectomy cases from a Japanese registry and showed that outcomes and prognostic variables differed by histology; in the sarcoma subgroup, shorter disease-free interval and increasing tumor burden were unfavorable signals [35]. Adachi et al. subsequently reported favorable postoperative survival in 38 patients, while cautioning against extrapolation in the presence of sarcoma histology, shorter disease-free interval, advanced primary stage, or synchronous extrapulmonary recurrence [36].

Recent illustrative reports remain consistent with this view, including the published report by Kodama et al., in which serial pulmonary metastasectomies supported long-term survival after uterine leiomyosarcoma recurrence [25].

Taken together, these data suggest that thoracic surgery is not a marginal adjunct but part of the broader surgical armamentarium for recurrent ULMS whenever disease distribution and physiology support a resection strategy [11,17,25,30,31,32,33,34,35,36].

#### 3.3.3. Peritoneal Dissemination and CRS/HIPEC-Type Strategies

Evidence for CRS/HIPEC in uterine sarcoma should be interpreted according to histology, because several reports combine ULMS with other uterine sarcoma subtypes and therefore cannot be directly extrapolated to recurrent ULMS alone [19,20,21,22].

The ULMS-specific systematic review by Matsuzaki et al. supports the feasibility of CRS/HIPEC in selected patients with disseminated peritoneal ULMS, but the included studies were small, mostly non-comparative, and associated with relevant grade ≥ 3 morbidity and perioperative mortality signals [19].

The multi-institutional study by Sardi et al. and the series by Díaz-Montes et al. support the feasibility of CRS/HIPEC-type approaches in uterine sarcoma with peritoneal dissemination, but their histologic heterogeneity limits direct inference for recurrent ULMS [20,21].

Ray et al. further reinforce that multimodal treatment for uterine sarcoma peritoneal sarcomatosis is feasible in specialized settings, although selection bias and histologic heterogeneity remain major interpretive limitations [22].

Therefore, CRS/HIPEC should be described as an individualized or investigational strategy for peritoneal-dominant disease in experienced centers, not as a routine extension of recurrent ULMS surgery [3,4,19,20,21,22].

#### 3.3.4. Published Illustrative Reports

Published single-patient and small-patient reports cannot establish effectiveness, but they are useful for showing the outer technical boundaries of what repeated surgery for recurrent ULMS can involve in real practice. In this review, they are treated exclusively as already published literature, not as new institutional clinical material; the most recent published reports identified in the update were Kodama et al. on serial pulmonary metastasectomy and Russo et al. on minimally invasive secondary cytoreduction for combined lung and pelvic relapse [25,26].

Zaránd et al. described a patient who underwent ten metastasectomies in thoracic and abdominal compartments and survived 23 years after the primary operation, thereby illustrating how long-term survival can occasionally coexist with iterative surgery across multiple body cavities [23].

Chetverikov et al. reported 13 interval debulking procedures for recurrent uterine sarcoma, including multiple bowel resections and peritonectomy, which demonstrates the extreme end of repeated compartment-based surgical salvage [24].

More recent illustrative reports have extended the same theme to serial pulmonary metastasectomy and minimally invasive secondary cytoreduction for mixed lung and pelvic recurrence, again reinforcing that recurrent ULMS may remain surgically actionable in exceptional patients when recurrence stays anatomically limited [25,26].

The previously published reports by Macciò et al. are retained only within this literature-review frame: one documented a prolonged minimally invasive surveillance-and-excision strategy in abdominal sarcomatosis from ULMS, whereas the other highlighted the surgical implications of abdominoperitoneal leiomyosarcomatosis after morcellation [27,28].

Illustrative published reports and highly selected experiences are summarized in Table 3 [23,24,25,26,27,28].

### 3.4. Selection Factors, Operative Boundaries, and Multidisciplinary Implications

Across the recurrent ULMS literature, complete gross resection is the most consistent operative objective associated with longer survival, but the practical meaning of resectability depends on more than lesion count alone [9,10,11,12,13,14,15,16,17].

Time to first recurrence is repeatedly important, with later relapse generally identifying a subgroup more likely to have indolent enough biology to justify iterative surgery [9,12,13,15,16].

Distribution matters as well: isolated or oligometastatic lung disease, compartmentalized abdominopelvic relapse, and anatomically limited extrapulmonary metastases are more compatible with a surgical strategy than rapidly progressive multi-organ dissemination [9,10,11,12,13,14,15,16,17,31,32,33,34,35,36].

Advanced imaging is helpful for operative planning, yet operative decision making still depends on whether the imaging pattern corresponds to a disease map that can realistically be cleared without disproportionate morbidity [3,4,7,11,12,13,14,15,16,17].

The surgeon must also anticipate when recurrent ULMS crosses conventional specialty boundaries, because bowel resection, splenectomy, diaphragm surgery, ureteral reimplantation, vascular control, and thoracic procedures may all become necessary components of complete resection in selected cases [7,8,9,10,11,12,13,14,15,16,17].

This is one reason why recurrent ULMS should be discussed in a multidisciplinary setting that includes gynecologic oncology, surgical oncology, thoracic surgery when thoracic or diaphragmatic extension is present, anesthesia, pathology, and medical oncology [3,4,7,8,15,16,17].

From a practical standpoint, the best candidates for repeated or extended cytoreduction are those in whom surgery is expected to convert visible disease to no gross residual disease, preserve acceptable postoperative function, and outperform the realistic alternative of non-curative systemic treatment alone [3,4,5,6,7,8,9,10,11,12,13,14,15,16,17].

These selection principles are summarized in Table 4 [3,7,8,9,10,11,12,13,14,15,16,17].

#### 3.4.1. Morbidity, Functional Recovery, and Quality-of-Life Implications

The decision to repeat cytoreduction should incorporate morbidity and functional cost, not only expected survival, because the procedures required to achieve complete gross resection may include bowel resection, urinary tract reconstruction, upper-abdominal dissection, diaphragmatic surgery, or thoracic metastasectomy [19,20,21,22,31,32,33,34,35,36].

In the multi-institutional series by Bizzarri et al., secondary cytoreduction frequently required bowel, bladder, or upper-abdominal procedures, yet no 30-day mortality was reported and the median time to postoperative chemotherapy was 41 days, suggesting that complex surgery can be delivered with acceptable early outcomes in tertiary referral settings [13].

These perioperative results should not be interpreted as evidence that repeated multivisceral surgery is broadly safe in all patients, because retrospective surgical cohorts preferentially include patients with adequate performance status, limited disease, and technically resectable recurrence [12,13,14,15,16,17].

For peritoneal-directed CRS/HIPEC-type strategies, ULMS-specific evidence remains sparse and associated with relevant grade ≥ 3 morbidity and perioperative mortality signals, supporting use only in highly selected cases rather than routine application [19,20,21,22].

Long-term functional outcomes, stoma rates, urinary morbidity, respiratory reserve after repeated thoracic surgery, return to baseline activity, and patient-reported quality of life are rarely captured in the available ULMS literature and should be included as core endpoints in future collaborative registries [19,20,21,22,29].

#### 3.4.2. Integration with Systemic Therapy and Treatment Sequencing

Systemic therapy and surgery should not be considered mutually exclusive in recurrent ULMS, because the clinical role of each modality depends on recurrence time, metastatic distribution, prior therapy, expected resectability, and patient fitness [3,4,5,6,15,17].

Systemic treatment is generally favored for rapidly progressive, multifocal, or unresectable disease, whereas surgery may be considered after multidisciplinary review when all visible disease can realistically be removed or when systemic therapy has converted disease to a stable and resectable pattern [3,4,5,6,12,15,17].

Contemporary systemic options for advanced or recurrent leiomyosarcoma include anthracycline-based regimens, gemcitabine/docetaxel-based therapy, trabectedin, pazopanib, dacarbazine, eribulin, and selected endocrine approaches for hormone receptor-positive tumors, with treatment choice influenced by previous therapy and patient tolerance [3,4,5,6,37].

The phase III LMS04 data support doxorubicin plus trabectedin followed by trabectedin maintenance as an important first-line option for metastatic or unresectable leiomyosarcoma in appropriate patients, but the higher toxicity of combination therapy must be considered when planning surgery or postoperative recovery [37].

After complete gross resection of recurrence, retrospective data do not consistently demonstrate an overall survival advantage from routine postoperative systemic therapy, so postoperative treatment should be individualized according to residual disease risk, prior systemic exposure, recurrence-free interval, morbidity, receptor status, and patient preference [5,6,12,15].

Immunotherapy is not established as a routine treatment for unselected recurrent ULMS and should be discussed mainly in the context of clinical trials or biomarker-defined tumor-agnostic indications [2,5,37,38,39,40].

The postoperative role of systemic therapy after secondary or repeated cytoreduction remains uncertain, because available studies are retrospective and postoperative treatment is strongly influenced by residual disease, prior therapy, recurrence-free interval, tumor burden, and postoperative recovery [5,6,9,10,11,12,13,14,15,16,17].

Cybulska et al. did not observe an OS advantage from adjuvant radiation, chemotherapy, or hormonal therapy after complete gross resection, although this finding should not be generalized because treatment allocation was not randomized [12].

Postoperative systemic therapy should therefore be individualized rather than automatic, with particular attention to residual disease status, prior systemic exposure, expected recovery, patient preference, receptor status, and availability of clinical trials [3,4,5,6,12,15,37,38,39,40,41].

#### 3.4.3. Biological Selection and Molecular Heterogeneity

ULMS is genomically heterogeneous, with recurrent alterations involving TP53, RB1, ATRX, PTEN, MED12, DNA damage response, cell-cycle regulation, and chromosomal instability; however, these alterations are not yet validated as criteria for surgical candidacy in recurrent disease [2,38,39,40,41].

At present, disease-free interval, recurrence pattern, lesion number, metastatic compartment, growth kinetics, and feasibility of complete gross resection remain more clinically actionable than molecular biomarkers for selecting patients for repeated cytoreduction [3,4,7,9,10,11,12,13,14,15,16,17].

Molecular profiling may still be relevant when it identifies targetable alterations, clarifies diagnosis, supports trial enrollment, or reveals biology that may influence systemic treatment sequencing before or after surgery [2,37,38,39,40,41].

Future registries should therefore integrate molecular data with operative variables and outcomes, because biological selection may partly explain why some patients experience serial resectable recurrences whereas others develop rapidly disseminated disease [2,15,16,17,29,38,39,40,41].

## 4. Discussion

The available literature converges on one central point: recurrent ULMS does not justify a uniform surgical or non-surgical rule, because the disease behaves very differently across patients and across recurrences in the same patient [1,2,3,4,5,6,7,8,9,10,11,12,13,14,15,16,17,23,24,25,26,27,28].

Instead, the most coherent reading of the evidence is that surgery retains value when recurrence remains anatomically mappable, technically resectable, and biologically slow enough for complete gross resection to be meaningful [3,4,7,8,9,10,11,12,13,14,15,16,17].

This interpretation is strengthened by the repetition of the same signal across independent retrospective datasets: outcomes are most favorable when no gross residual disease is achieved, when recurrence-free interval is longer, and when disease remains limited to one or a few compartments [9,10,11,12,13,14,15,16,17].

At the same time, the literature does not support an indiscriminate enthusiasm for aggressive surgery, because none of the available series can fully disentangle operative benefit from favorable selection [7,8,9,10,11,12,13,14,15,16,17].

That limitation is especially relevant when recurrent ULMS requires multivisceral resection, since bowel surgery, urinary reconstruction, upper-abdominal dissection, diaphragmatic resection, thoracic procedures, and occasionally peritoneal-directed strategies may all be technically justified in selected patients while still being inappropriate in others with rapidly progressive biology [7,8,9,10,11,12,13,14,15,16,17,19,20,21,22,23,24,25,26].

The published illustrative reports are useful precisely because they show the same tension at the margins: repeated surgery can remain feasible over years in exceptional patients, but these reports do not eliminate selection bias and should not be extrapolated to biologically uncontrolled disease [23,24,25,26,27,28].

These reports and the cohort literature also highlight that the threshold between gynecologic oncology and other surgical disciplines becomes increasingly permeable as recurrent ULMS extends into the upper abdomen, retroperitoneum, diaphragm, peritoneal cavity, and thorax [7,8,11,12,13,14,15,16,17,19,20,21,22,31,32,33,34,35,36].

From a practical viewpoint, upper-abdominal, diaphragmatic, bowel, urinary tract, and peritoneal procedures represent the main technical extensions of abdominopelvic cytoreduction; thoracic input may be relevant only in selected cases involving pulmonary metastasectomy or complex diaphragmatic/pleural management [7,11,15,17,31,32,33,34,35,36].

Modern imaging should be viewed as an enabler rather than a substitute for operative judgment, because PET/CT can identify dominant hypermetabolic disease and interval progression, yet the true operative burden may only become evident during meticulous compartment-based exploration [3,4,15,17].

Published illustrative reports of repeated metastasectomy are particularly helpful in this respect, because they show that long-term survivorship can emerge from serial operations performed over years and across compartments, even though such experiences should never be mistaken for proof that the same strategy should be generalized [23,24,25,26,27,28].

For the field as a whole, the unmet need is therefore methodological rather than conceptual: collaborative datasets should report recurrence pattern, operative complexity, residual disease status, site-specific procedures, postoperative morbidity, patient-reported recovery, and the sequencing of systemic therapy so that future selection criteria are based on more than anecdotal success; as of 14 May 2026, the literature still lacks trial-level evidence and remains anchored to retrospective cohorts, systematic reviews of small noncomparative experiences, and highly selected illustrative reports [3,4,7,13,14,15,16,17,22,29].

### 4.1. Selection Bias and Interpretation of Surgical Benefit

The principal limitation of the recurrent ULMS surgical literature is confounding by indication, because patients selected for surgery usually have more favorable disease biology, lower metastatic burden, longer disease-free interval, better performance status, and a higher probability of complete gross resection than patients managed non-surgically [7,9].

Complete gross resection is therefore both a therapeutic endpoint and a surrogate marker of favorable anatomy and tumor biology, making it difficult to separate the effect of surgery from the biology that made surgery possible [10,11,12,13,14].

Survival differences favoring surgical cohorts should consequently be interpreted as association signals rather than causal proof, particularly when non-operated patients include those with rapidly progressive, multifocal, or unresectable disease [16,17].

The most defensible conclusion is that repeated cytoreduction may be valuable in selected patients, not that surgery should be generalized to all patients with recurrent ULMS [3,4,7,9,15].

### 4.2. Future Directions

Future work should move beyond isolated anecdotal success toward multicenter prospective registries and more standardized reporting of recurrence pattern, operative burden, residual disease, postoperative recovery, thoracic or peritoneal-directed local therapies, and systemic therapy sequencing, so that the real boundaries of extended cytoreductive surgery in recurrent ULMS can finally be defined [3,4,7,13,14,15,16,17,29].

## 5. Conclusions

The available literature supports a cautious role for repeated or extended cytoreductive surgery in recurrent ULMS when recurrence is anatomically limited, complete gross resection appears realistic, and the expected functional cost is acceptable. This conclusion rests on retrospective association signals rather than prospective comparative evidence, and it must therefore be interpreted through the lens of selection bias [3,4,7,8,9,10,11,12,13,14,15,16,17,19,20,21,22,25,26,34,35,36].

In selected patients, iterative metastasectomy or multivisceral cytoreduction may include thoracic, upper-abdominal, bowel, urinary tract, diaphragmatic, or peritoneal-directed procedures, but escalation should remain individualized, multidisciplinary, and explicitly linked to the likelihood of no gross residual disease [7,8,9,10,11,12,13,14,15,16,17,19,20,21,22,23,24,25,26,31,32,33,34,35,36].

Future collaborative registries should capture disease-free interval, recurrence site and burden, operative procedures, residual disease status, morbidity, recovery, systemic-therapy sequencing, survival, and patient-reported outcomes, including patients considered for surgery but ultimately not operated on [13,14,15,16,17,29].

## Figures and Tables

**Table 1:** Guideline-based principles relevant to surgery for recurrent uterine leiomyosarcoma and their relationship with the available retrospective evidence.

Guideline Principle	Practical Implication	Interpretation
Management should be multidisciplinary and histology-specific [3,4]	Recurrent ULMS should be discussed in a sarcoma/gynecologic oncology MDT	Supports individualized decisions rather than uniform surgical rules
Complete resection is the surgical goal when disease is resectable [3,4]	Surgery should be considered only if no gross residual disease appears realistic	Aligns with retrospective surgical cohorts
Recurrence management depends on site, interval, prior treatment, symptoms, and patient status [3,4]	DFI, recurrence compartment, metastatic burden, and fitness should be reported	Supports the proposed selection framework
Systemic therapy remains central for unresectable or progressive metastatic disease [3,4,5,6]	Surgery should not delay appropriate systemic therapy in rapidly progressive disease	Avoids overstatement of surgical benefit
Radiotherapy may be considered as local treatment in selected settings [3,4]	RT may complement or substitute surgery for local control in selected patients	Should not be conflated with systemic disease control
Evidence remains low-level for repeated cytoreduction and CRS/HIPEC [3,4,19,20,21,22]	Registry-level prospective data are needed	Supports cautious conclusions

Notes: CRS, cytoreductive surgery; DFI, disease-free interval; HIPEC, hyperthermic intraperitoneal chemotherapy; MDT, multidisciplinary team; RT, radiotherapy; ULMS, uterine leiomyosarcoma.

**Table 2:** Representative cohort evidence and lightweight structured appraisal of surgical cytoreduction/metastasectomy in metastatic or recurrent ULMS.

Main Limitation	Morbidity	Follow-Up/Time Horizon and Survival Outcome	CGR/Residual Disease	Selection Features	Sites/Setting	N	Design/Population	Study
Retrospective selection; older treatment era	NR	Median DSS from recurrence 1.8 years; SCS, longer TTR, localized recurrence independently associated with improved DSS	NR	Longer time to recurrence, localized recurrence	Recurrent ULMS	128	Retrospective recurrent ULMS cohort	Giuntoli et al. [9]
Initial metastatic rather than recurrent-only setting	Morbidity not quantified in abstract; authors emphasize weighing morbidity	PFS 14.2 vs. 6.8 months; OS 31.9 vs. 20.2 months for CGR vs. residual disease	CGR 41/84 surgically managed patients	Surgical candidates selected for cytoreduction	Intraperitoneal/extraperitoneal metastatic disease	96	Retrospective metastatic-at-diagnosis ULMS	Leitao et al. [10]
Single-center retrospective cohort	NR	2-year DSS 71.2%; TTR and optimal resection associated with survival	Residual disease available in 37	Surgical resection at first recurrence	17 pelvic, 18 distant, 6 both; thoracic procedure alone in 13	41	Retrospective recurrent ULMS resection cohort	Leitao et al. [11]
Highly selected surgical cohort	NR	Median OS 37.7 months for abdominal/pelvic recurrence; 78.1 months for lung-only recurrence	CGR 58/62	No prior therapy for recurrence; high probability of CGR	29 abdominopelvic, 30 lung-only, 3 both	62	Retrospective first-recurrence ULMS cohort	Cybulska et al. [12]
Surgical cohort only; no randomized comparator	No 30-day mortality; four major postoperative complications; median hospital stay 7 days	Median RFS 16.0 months; 5-year OS 76%	No residual tumor 35/38	Tertiary centers; secondary cytoreduction	Isolated disease in 44.7%; bowel/bladder/upper abdomen frequently involved	38	Multi-institutional recurrent ULMS surgical cohort	Bizzarri et al. [13]
Not ULMS-specific	NR	Reported benefit signal for SCS	NR	Secondary cytoreduction	Mixed uterine sarcoma	NR	Mixed recurrent uterine sarcoma cohort	Nakamura et al. [14]
Retrospective single-center selection	NR	5-year OS 62.0% vs. 28.0% for SCS vs. non-SCS; early recurrence <12 months adverse	Complete resection 47/52	First recurrence; operability	SCS *n* = 52; systemic therapy *n* = 19; thoracic surgery in 10 lung-only recurrences	71	Retrospective first-recurrence ULMS cohort	Yuan et al. [15]
Small cohort; cytoreduction benefit not statistically significant	NR	OS 19 vs. 15 months for CRS + CT vs. CT only; recurrence >6 months associated with better OS	NR	Recurrence timing emphasized	CRS + CT vs. CT after recurrence	59 total; 44 recurrences	Retrospective recurrent ULMS cohort	Tunç et al. [16]

Notes: ULMS, uterine leiomyosarcoma; CGR, complete gross resection; CRS, cytoreductive surgery; CT, chemotherapy; DSS, disease-specific survival; NR, not reported; OS, overall survival; PFS, progression-free survival; RFS, recurrence-free survival; SCS, secondary cytoreductive surgery; TTR, time to recurrence. Reported follow-up or survival time horizon was extracted when available and was not imputed when not reported.

**Table 3:** Illustrative published reports of repeated or extensive surgery for recurrent uterine leiomyosarcoma/uterine sarcoma.

Illustrative Report	Why It Matters
Zaránd et al., 2016 [23]	Ten metastasectomies across thoracic and abdominal compartments showed that iterative metastasectomy can occasionally coexist with very long survival.
Chetverikov et al., 2021 [24]	Thirteen interval debulking operations, including bowel resections and peritonectomy, illustrate the extreme technical end of repeat surgical salvage.
Kodama et al., 2025 [25]	Serial pulmonary metastasectomies support the concept of repeated thoracic surgery in lung-limited recurrent ULMS.
Russo et al., 2026 [26]	Minimally invasive secondary cytoreduction shows that selected recurrent patterns may still be amenable to laparoscopy/robotics.
Macciò et al., 2016 [27]	Repeated laparoscopic surveillance and excision demonstrated a prolonged minimally invasive strategy in abdominal sarcomatosis from ULMS.
Macciò et al., 2017 [28]	Abdominal dissemination after morcellation highlighted the surgical implications of recurrent intraperitoneal disease.

Notes: ULMS, uterine leiomyosarcoma.

**Table 4:** Literature-based practical variables that support or weaken consideration of repeated/extended cytoreductive surgery in recurrent ULMS.

Practical Variable	Interpretation for Recurrent ULMS Surgery
Longer recurrence-free interval [9,12,13,15,16]	Usually favors surgery because it suggests less explosive biology.
Compartmentalized or isolated recurrence [9,10,11,12,13,14,15,16,17]	Usually favors surgery because complete gross resection is more plausible.
High probability of no gross residual disease [9,10,11,12,13,14,15,16,17]	Central criterion supporting cytoreductive intent.
Lung-limited or oligometastatic thoracic disease [11,12,17,30,31,32,33,34,35,36]	May justify pulmonary metastasectomy or staged thoracic surgery.
Need for multivisceral resection [7,8,9,10,11,12,13,14,15,16,17]	Does not automatically preclude surgery, but demands experienced multidisciplinary planning.
Diffuse rapidly progressive dissemination [3,4,7,8,9,10,11,12,13,14,15,16,17]	Usually weakens the rationale for aggressive cytoreduction.

Notes: ULMS, uterine leiomyosarcoma.

## Data Availability

No new datasets were generated or analyzed in this narrative review. All cited data derive from previously published sources.

## Abbreviations

The following abbreviations are used in this manuscript:

CGR	complete gross resection
CRS	cytoreductive surgery
DFI	disease-free interval
HIPEC	hyperthermic intraperitoneal chemotherapy
PET/CT	positron emission tomography/computed tomography
ULMS	uterine leiomyosarcoma
TP53	tumor protein 53
RB1	RB transcriptional corepressor 1
ATRX	alpha thalassemia retardation syndrome X-linked chromatin remodeler
PTEN	phosphatase and tensin homolog
MED12	mediator complex subunit 12
DNA	deoxyribonucleic acid

## References

[1] George S., Serrano C., Hensley M.L., Ray-Coquard I. (2018). Soft Tissue and Uterine Leiomyosarcoma. J. Clin. Oncol..

[2] Yang Q., Madueke-Laveaux O.S., Cun H., Wlodarczyk M., Garcia N., Carvalho K.C., Al-Hendy A. (2024). Comprehensive Review of Uterine Leiomyosarcoma: Pathogenesis, Diagnosis, Prognosis, and Targeted Therapy. Cells.

[3] Ray-Coquard I., Casali P.G., Croce S., Fennessy F.M., Fischerova D., Jones R., Sanfilippo R., Zapardiel I., Amant F., Blay J.-Y. (2024). ESGO/EURACAN/GCIG guidelines for the management of patients with uterine sarcomas. Int. J. Gynecol. Cancer.

[4] Pérez-Fidalgo J.A., Martín-Broto J., Ortega E., Ponce J., Redondo A., Sevilla I., Valverde C., Verdum J.I., López M.G., Sebio A. (2023). Uterine sarcomas: Clinical practice guidelines for diagnosis, treatment, and follow-up by the Spanish Group for Research on Sarcomas (GEIS). Ther. Adv. Med. Oncol..

[5] Arend R.C., Toboni M.D., Montgomery A.M., Burger R.A., Olawaiye A.B., Monk B.J., Herzog T.J. (2018). Systemic Treatment of Metastatic/Recurrent Uterine Leiomyosarcoma: A Changing Paradigm. Oncologist.

[6] Amant F., Lorusso D., Mustea A., Duffaud F., Pautier P. (2015). Management Strategies in Advanced Uterine Leiomyosarcoma: Focus on Trabectedin. Sarcoma.

[7] Ghirardi V., Bizzarri N., Guida F., Vascone C., Costantini B., Scambia G., Fagotti A. (2019). Role of surgery in gynaecological sarcomas. Oncotarget.

[8] Korets S.B., Curtin J.P. (2012). Surgical Options for Recurrent Uterine Sarcomas. Am. Soc. Clin. Oncol. Educ. Book.

[9] Giuntoli R.L., Garrett-Mayer E., Bristow R.E., Gostout B.S. (2007). Secondary cytoreduction in the management of recurrent uterine leiomyosarcoma. Gynecol. Oncol..

[10] Leitao M.M., Zivanovic O., Chi D.S., Hensley M.L., O’Cearbhaill R., Soslow R.A., Barakat R.R. (2012). Surgical cytoreduction in patients with metastatic uterine leiomyosarcoma at the time of initial diagnosis. Gynecol. Oncol..

[11] Leitao M.M., Brennan M.F., Hensley M., Sonoda Y., Hummer A., Bhaskaran D., Venkatraman E., Alektiar K., Barakat R.R. (2002). Surgical Resection of Pulmonary and Extrapulmonary Recurrences of Uterine Leiomyosarcoma. Gynecol. Oncol..

[12] Cybulska P., Sioulas V., Orfanelli T., Zivanovic O., Mueller J.J., Broach V.A., Roche K.C.L., Sonoda Y., Hensley M.L., O’Cearbhaill R.E. (2019). Secondary surgical resection for patients with recurrent uterine leiomyosarcoma. Gynecol. Oncol..

[13] Bizzarri N., Ghirardi V., Di Fiore G.L.M., De Iaco P., Gadducci A., Casarin J., Perrone A.M., Pasciuto T., Scambia G., Fagotti A. (2019). Secondary cytoreductive surgery in recurrent uterine leiomyosarcoma: A multi-institutional study. Int. J. Gynecol. Cancer.

[14] Nakamura K., Kajiyama H., Utsumi F., Suzuki S., Niimi K., Sekiya R., Sakata J., Yamamoto E., Shibata K., Kikkawa F. (2018). Secondary cytoreductive surgery potentially improves the oncological outcomes of patients with recurrent uterine sarcomas. Mol. Clin. Oncol..

[15] Yuan H., Wang Y., Li N., Wu L., Yao H. (2024). Clinical characteristics and treatment outcomes of women with recurrent uterine leiomyosarcoma. Orphanet J. Rare Dis..

[16] Tunç M., Akıllı H., Kuşçu E., Ayhan A. (2025). Timing and survival benefits of cytoreduction in patients with recurrent leiomyosarcoma. Arch. Gynecol. Obstet..

[17] Delisle M., Alshamsan B., Nagaratnam K., Smith D., Wang Y., Srikanthan A. (2022). Metastasectomy in Leiomyosarcoma: A Systematic Review and Pooled Survival Analysis. Cancers.

[18] Baethge C., Goldbeck-Wood S., Mertens S. (2019). SANRA-a scale for the quality assessment of narrative review articles. Res. Integr. Peer Rev..

[19] Matsuzaki S., Matsuzaki S., Chang E.J., Yasukawa M., Roman L.D., Matsuo K. (2021). Surgical and oncologic outcomes of hyperthermic intraperitoneal chemotherapy for uterine leiomyosarcoma: A systematic review of literature. Gynecol. Oncol..

[20] Sardi A., Sipok A., Baratti D., Deraco M., Sugarbaker P., Salti G., Yonemura Y., Sammartino P., Glehen O., Bakrin N. (2017). Multi-institutional study of peritoneal sarcomatosis from uterine sarcoma treated with cytoreductive surgery and hyperthermic intraperitoneal chemotherapy. Eur. J. Surg. Oncol..

[21] Díaz-Montes T.P., El-Sharkawy F., Lynam S., Harper A., Sittig M., MacDonald R., Gushchin V., Sardi A. (2018). Efficacy of Hyperthermic Intraperitoneal Chemotherapy and Cytoreductive Surgery in the Treatment of Recurrent Uterine Sarcoma. Int. J. Gynecol. Cancer.

[22] Ray M.D., Kapoor R., Bn N. (2025). Multimodal management of peritoneal sarcomatosis in uterine sarcoma: Long term outcomes from a single institute in India. Eur. J. Obstet. Gynecol. Reprod. Biol..

[23] Zaránd A., Jósa V., Vass T., Baranyai Z. (2016). Long-term survival of a patient with uterine leiomyosarcoma treated with repeated metastasectomies: A case report. Ann. Clin. Case Rep..

[24] Chetverikov S., Maksymovskyi V., Atanasov D., Chetverikov M., Chetverikova-Ovchynnyk V. (2021). Multiple Interval Debulking Surgery in Recurrent Uterine Sarcoma (Case Report). Georgian Med. News.

[25] Kodama K., Momozane T., Takehara H., Sato K. (2025). Long-term survival following serial pulmonary metastasectomies for uterine leiomyosarcoma: A case report. Gen. Thorac. Cardiovasc. Surg. Cases.

[26] Russo S.A., Diella C., Certelli C., Fanfani F., Hudry D.J., Avesani G., Lococo F., Gallotta V. (2025). Minimally invasive secondary cytoreduction for lung and pelvic recurrence in uterine leiomyosarcoma. Int. J. Gynecol. Cancer.

[27] Macciò A., Kotsonis P., Chiappe G., Melis L., Zamboni F., Madeddu C. (2016). Long-Term Survival in a Patient With Abdominal Sarcomatosis From Uterine Leiomyosarcoma: Role of Repeated Laparoscopic Surgery in Treatment and Follow-Up. J. Minim. Invasive Gynecol..

[28] Macciò A., Chiappe G., Kotsonis P., Lavra F., Serra M., Demontis R., Madeddu C. (2017). Abdominal leiomyosarcomatosis after surgery with external morcellation for occult smooth muscle tumors of uncertain malignant potential. Int. J. Surg. Case Rep..

[29] Bacalbasa N., Balescu I., Dima S., Brasoveanu V., Popescu I. (2015). Prognostic factors and survival in patients treated surgically for primary and recurrent uterine leiomyosarcoma: A single center experience. Anticancer. Res..

[30] Istl A.C., Desravines N., Nudotor R., Stone R., Greer J.B., Meyer C.F., Johnston F.M. (2023). Treatment patterns and outcomes for primary uterine leiomyosarcoma with synchronous isolated lung metastases: A National Cancer Database study of primary resection and metastasectomy. Gynecol. Oncol. Rep..

[31] Paik E.S., Yoon A., Lee Y.-Y., Kim T.-J., Lee J.-W., Bae D.-S., Kim B.-G. (2015). Pulmonary metastasectomy in uterine malignancy: Outcomes and prognostic factors. J. Gynecol. Oncol..

[32] Stork T., Hegedüs B., Guder W., Hamacher R., Hardes J., Kaths M., Plönes T., Pöttgen C., Schildhaus H.-U., Streitbürger A. (2022). Prognostic Factors for Leiomyosarcoma with Isolated Metastases to the Lungs: Impact of Metastasectomy. Ann. Surg. Oncol..

[33] Burt B.M., Ocejo S., Mery C.M., Dasilva M., Bueno R., Sugarbaker D.J., Jaklitsch M.T. (2011). Repeated and Aggressive Pulmonary Resections for Leiomyosarcoma Metastases Extends Survival. Ann. Thorac. Surg..

[34] Kanzaki R., Susaki Y., Takami K., Funakoshi Y., Sakamaki Y., Kodama K., Yokouchi H., Ikeda N., Kadota Y., Thoracic Surgery Study Group of Osaka University (TSSGO) (2020). Long-Term Outcomes of Pulmonary Metastasectomy for Uterine Malignancies: A Multi-institutional Study in the Current Era. Ann. Surg. Oncol..

[35] Nobori Y., Anraku M., Yamauchi Y., Mun M., Yoshino I., Nakajima J., Ikeda N., Matsuguma H., Iwata T., Shintani Y. (2023). Risk-adjusted hazard analysis of survival after pulmonary metastasectomy for uterine malignancies in 319 cases. JTCVS Open.

[36] Adachi H., Ito H., Nagashima T., Isaka T., Murakami K., Kikunishi N., Shigeta N., Saito A. (2025). Prognostic Impact of Pulmonary Metastasectomy for Uterine Malignancies: A Retrospective Analysis of 38 Cases. Cancers.

[37] Pautier P., Italiano A., Piperno-Neumann S., Chevreau C., Penel N., Firmin N., Boudou-Rouquette P., Bertucci F., Lebrun-Ly V., Ray-Coquard I. (2024). Doxorubicin–Trabectedin with Trabectedin Maintenance in Leiomyosarcoma. N. Engl. J. Med..

[38] Chudasama P., Mughal S.S., Sanders M.A., Hübschmann D., Chung I., Deeg K.I., Wong S.-H., Rabe S., Hlevnjak M., Zapatka M. (2018). Integrative genomic and transcriptomic analysis of leiomyosarcoma. Nat. Commun..

[39] Dall G.V., Hamilton A., Ratnayake G., Scott C., Barker H. (2022). Interrogating the Genomic Landscape of Uterine Leiomyosarcoma: A Potential for Patient Benefit. Cancers.

[40] Momeni-Boroujeni A., Yousefi E., Balakrishnan R., Riviere S., Kertowidjojo E., Hensley M.L., Ladanyi M., Ellenson L.H., Chiang S. (2023). Molecular-Based Immunohistochemical Algorithm for Uterine Leiomyosarcoma Diagnosis. Mod. Pathol..

[41] Denu R.A., Dann A.M., Keung E.Z., Nakazawa M.S., Haddad E.F.N. (2024). The Future of Targeted Therapy for Leiomyosarcoma. Cancers.

